# Associations between air temperature and cardio-respiratory mortality in the urban area of Beijing, China: a time-series analysis

**DOI:** 10.1186/1476-069X-10-51

**Published:** 2011-05-25

**Authors:** Liqun Liu, Susanne Breitner, Xiaochuan Pan, Ulrich Franck, Arne Marian Leitte, Alfred Wiedensohler, Stephanie von Klot, H-Erich Wichmann, Annette Peters, Alexandra Schneider

**Affiliations:** 1 Helmholtz Zentrum Muenchen, German Research Center for Environmental Health, Institute of Epidemiology II, Neuherberg, Germany; 2 Ludwig-Maximilians-University Munich, IBE Chair of Epidemiology, Munich, Germany; 3 Peking University Health Science Center, School of Public Health, Beijing, China; 4 Helmholtz Centre for Environmental Research - UFZ, Core Facility Studies, Leipzig, Germany; 5 Physics Department, Leibniz Institute for Tropospheric Research (IfT), Leipzig, Germany; 6 Helmholtz Zentrum Muenchen, German Research Center for Environmental Health, Institute of Epidemiology I, Neuherberg, Germany

## Abstract

**Background:**

Associations between air temperature and mortality have been consistently observed in Europe and the United States; however, there is a lack of studies for Asian countries. Our study investigated the association between air temperature and cardio-respiratory mortality in the urban area of Beijing, China.

**Methods:**

Death counts for cardiovascular and respiratory diseases for adult residents (≥15 years), meteorological parameters and concentrations of particulate air pollution were obtained from January 2003 to August 2005. The effects of two-day and 15-day average temperatures were estimated by Poisson regression models, controlling for time trend, relative humidity and other confounders if necessary. Effects were explored for warm (April to September) and cold periods (October to March) separately. The lagged effects of daily temperature were investigated by polynomial distributed lag (PDL) models.

**Results:**

We observed a J-shaped exposure-response function only for 15-day average temperature and respiratory mortality in the warm period, with 21.3°C as the threshold temperature. All other exposure-response functions could be considered as linear. In the warm period, a 5°C increase of two-day average temperature was associated with a RR of 1.098 (95% confidence interval (95%CI): 1.057-1.140) for cardiovascular and 1.134 (95%CI: 1.050-1.224) for respiratory mortality; a 5°C decrease of 15-day average temperature was associated with a RR of 1.040 (95%CI: 0.990-1.093) for cardiovascular mortality. In the cold period, a 5°C increase of two-day average temperature was associated with a RR of 1.149 (95%CI: 1.078-1.224) for respiratory mortality; a 5°C decrease of 15-day average temperature was associated with a RR of 1.057 (95%CI: 1.022-1.094) for cardiovascular mortality. The effects remained robust after considering particles as additional confounders.

**Conclusions:**

Both increases and decreases in air temperature are associated with an increased risk of cardiovascular mortality. The effects of heat were immediate while the ones of cold became predominant with longer time lags. Increases in air temperature are also associated with an immediate increased risk of respiratory mortality.

## Background

In recent years, concern on the effects of meteorological factors on population health has increased. Research started by exploring the effects of weather, since its relationship with certain health outcomes is relatively easy to be investigated compared to rather long-term climate changes. The Intergovernmental Panel on Climate Change (IPCC) has recommended short-term air temperature fluctuations as one of the main markers for analyzing the association between climate and mortality or morbidity [1].

So far, the association between air temperature and mortality has been investigated in various locations of the world, either by simple descriptive statistics or by time-series or case-crossover approaches. Results obtained from heat wave events formed most of the existing evidence of heat effects on mortality from 1970s until today [2]. The 2003 European heat wave and 2006 California heat wave are two prominent recent events. Excess deaths in early August 2003 were speculated to be at least 33,120 for Western Europe [3] (maximum "% excess death" was found to be 60% for France from 1 to 20 August, 2003 [4]); while 655 (6%) excess deaths were estimated for California from 15 July to 1 August, 2006 [5]. However, not only heat waves but also increases in moderate temperature contribute to the observed heat-related mortality. Exposure-response functions between mortality time-series and continuous temperature measures have shown V-, U- or J-shaped associations, and the range of temperature corresponding with a minimum mortality ("threshold", "turning point" or "optimum temperature") was reported to be related with latitude [6,7]. The residents of lower latitudes tended to be more vulnerable only at higher temperature values, indicating less susceptibility to heat [8-10].

Excess winter mortality has been well known (which may have also caused that in recent years, particularly in the light of global warming, there were fewer studies particularly focusing on cold spells or temperature decreases). The Eurowinter study [11] found that annual excess deaths due to cold ranged from 408 to 1,617 for eight European regions on days colder than 18°C. Barnett et al. [12] compared coronary events occurring in the coldest 25% of periods with those occurring in the rest of periods among the WHO MONICA project population and found an overall increase. In a recent large multi-centre European study (PHEWE, 15 cities), Analitis et al. [13] found that a 1°C decrease in 16-day-average minimum apparent temperature was associated with 1.25%-3.30% increases of total or cause-specific mortalities.

Until today, most studies regarding weather and climate effects on health have been conducted in Europe and the United States; however, there is a lack of data and publications about the temperature-mortality relationship in the Asian region. For this reason, we conducted the present study aiming at investigating the association between daily air temperature and daily cardiovascular as well as respiratory mortality in the urban area of Beijing, China. Moreover, we were interested in the age-group that is affected the most by heat or cold in this area and investigated in addition, if air pollution plays a role in the temperature-mortality relationship.

## Material and methods

### Study area and period

**Figure 1:**
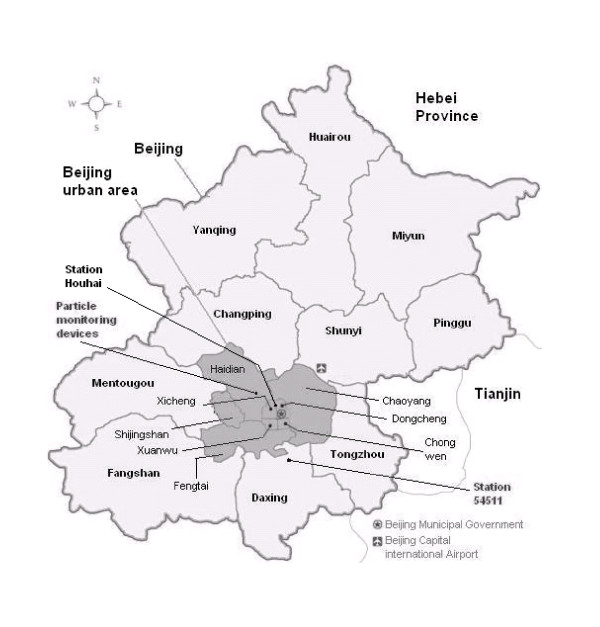
**Beijing and the urban area of Beijing (shaded)**.

We conducted the study in the urban area of Beijing, China, from 1 Jan 2003 to 31 Aug 2005 (974 days). Beijing is located on the North China Plain surrounded by mountains of 1000-1500 m in altitude to the west, north, and northeast, while Bohai Sea on the southeast side. Typical warm temperate semi-humid continental monsoon climate brings Beijing hot, humid summers and cold, dry winters. Springs and autumns are both of relatively short duration. The urban area of Beijing is about 1,368 km^2 ^consisting of eight districts (see Figure 1) with approximately 7,072,000 registered permanent residents [14].

### Mortality data

We obtained mortality data in the Beijing urban area for adult residents (≥ 15 years) from Beijing Centers for Diseases Control and Prevention (CDC). We calculated daily death counts for adults (referring as "the whole population") as well as for individuals of 65 years and older. Daily death counts included deaths due to cardiovascular (ICD-10 code: I00-I99), respiratory (J00-J99), and cardiorespiratory (I00-J99) diseases. We further considered death counts for ischemic heart diseases (I20-I25) and cerebrovascular diseases (I60-I69), which were the two major cardiovascular subcategories. Influenza and pneumonia (J10-J18) and chronic lower respiratory diseases (J40-J47), the two major respiratory subcategories, were not analyzed because of too small counts.

### Meteorological and air pollution data

Daily meteorological data were available from China Meteorological Data Sharing Service System (station 54511, located at N39°48' E116°28' in the south eastern part of Beijing within Daxing District, see Figure 1) and included daily mean temperature, relative humidity, and barometric pressure. We further calculated apparent temperature (a measure of individually perceived discomfort due to a combination of temperature and humidity) [15]. Daily mean meteorological data from another measurement station (Houhai, located in the centre of Beijing, see Figure 1) was gathered from an internet weather service (Weather Underground 2011) as well, but contained missing values. The Pearson correlation coefficients for valid days between the two data sources were 0.995, 0.967 and 0.999 for daily air temperature, relative humidity and barometric pressure, respectively, indicating a good agreement.

Daily mass concentrations of ambient particulate matter with an aerodynamic diameter <2.5 μm (PM_2.5_) and number concentrations of ambient particles with an aerodynamic diameter <0.1 μm (ultrafine particles, UFP) were obtained from a joint cooperation between Peking University, Beijing, China, and Leibniz-Institute for Tropospheric Research, Leipzig, Germany [16]. The measurement station for aerosol size distribution data is located on Peking University campus area in the north western part of Beijing (see Figure 1). The number size distribution was used to calculate number concentrations of UFP, and mass concentrations of PM_2.5 _assuming spherical particles with a mean particle density of 1.5 g cm^-3^. Details are described elsewhere [17]. Particle data were available only from March 2004 on.

### Statistical analyses

We used generalized semi-parametric Poisson regression to model the natural logarithm of the expected daily death counts as a function of the predictor variables. Penalized splines were used to allow for non-linear confounding and temperature effects. Data were analyzed using the package "mgcv" version 1.4-1.1 in the statistical software R version 2.7.2 (R Development Core Team, 2008).

We explored the effects of air temperature on mortality within warm period (April to September) and cold period (October to March) separately.

In a first step, a base model was built without air temperature exposure for each category of mortality individually (see Additional file 1, Table S1). To control systematic variations over time, we considered long-term trend as well as dummy variables for season, day of the week (DOW), and public holidays as potential confounders. As potential meteorological confounders we considered daily mean relative humidity and barometric pressure with the same type of lag as the temperature term. To ensure sufficient adjustment for season and other meteorological parameters, time trend and relative humidity were forced into all models. Season, day of the week, public holidays and barometric pressure were only included if they improved model fit. As a criterion to guide the selection of degrees of freedom (DF) for trend, we used the minimization of the absolute value of the sum of the partial autocorrelation function (PACF) of the model's residuals for a fixed number of lags [18]. Model selection for the other confounders was carried out by minimizing the Generalized Cross Validation (GCV) criterion [19].

We considered the mean of lags 0 to 1 and of lags 0 to 14 for air temperature exposure. The focus on these averages was chosen on the basis of previous studies conducted in Europe, Northern America, and other places around the world [13,20-22]. Firstly, we added them to the base models and estimated the exposure-response functions for temperature effects using penalized splines with four knots. Then, if the function was linear or almost linear, temperature effects were directly presented as relative risk (RR) of death per 5°C increase if positive linear or decrease if negative linear, respectively. If the function was non-linear, we selected a temperature breakpoint (which we commonly call "threshold") by minimizing the Akaike Information Criterion (AIC) for a range of different threshold values. Then for a J-shaped function, only the temperatures above the threshold were used for effect estimation. In this case, temperature effects were presented as relative risk (RR) of death per 5°C increase in temperature above the threshold.

After having explored the effects of air temperature on mortality for the whole population as described above, we repeated the same procedure for mortality of elderly people (65 years and above) only.

Furthermore, we applied polynomial distributed lag (PDL) models [23] to avoid problems related to co-linearity among lagged exposure variables. We investigated the lagged effects of air temperature up to 29 days on the whole population as well as the elderly people (65 years and above), for warm period and cold period. We constrained the shape of the distributed lag curve to follow a polynomial of 5^th^-order in order to get a flexible functional form.

### Sensitivity analyses

We used different threshold temperatures for a J-shaped function for the whole population and the elderly people. All other sensitivity analyses were done only for the whole population. Sensitivity analyses included the use of different values of smoothness for the functions of time trend. We also estimated the exposure-response functions using apparent temperature instead of mean air temperature, again considering two-day and 15-day averages. Moreover, we re-analyzed the air temperature effects on mortality for the shorter warm (April to September 2004 plus April to August 2005) or cold (March 2004 plus October 2004 to March 2005) period, during which the ambient particle data was available. We then also included the concentrations of PM_2.5 _or UFP linearly as additional adjustments using a lag of two days, as this seemed to be the most appropriate lag for the association between air pollution and mortality (Breitner S et al. Unpublished work).

## Results

### Mortality data

There were 14,723 cardiovascular and 3,150 respiratory deaths in the warm period, while 17,493 and 4,007 in the cold period. Table 1 presents descriptive statistics for daily death counts by cause and age groups, for warm and cold periods. Deaths occurred within individuals of 65 years and older were 83% to 88% of all cases due to each cause. Daily death counts followed a seasonal pattern with peaks in winters and troughs in summers in the whole population as well as the aged people, while daily death counts for the group of 15 to 64 years had no obvious seasonal pattern (see Additional file 1, Figure S1).

**Table 1:** Descriptive statistics of daily death counts in the urban area of Beijing by time period, age group, and cause of death

	Whole population								65+ years							
	Warm period				Cold period				Warm period				Cold period			
	
Cause of death (ICD-10 code)	Mean ± SD	Min	Median	Max	Mean ± SD	Min	Median	Max	Mean ± SD	Min	Median	Max	Mean ± SD	Min	Median	Max
Cardiovascular diseases (I00-I99)	28 ± 9	8	29	51	38 ± 8	17	38	70	24 ± 7	6	24	44	32 ± 7	13	32	62
Respiratory diseases (J00-J99)	6 ± 3	0	6	17	9 ± 4	0	8	25	5 ± 3	0	5	15	8 ± 3	0	8	22
Ischemic heart diseases (I20-I25)	12 ± 4	1	12	26	16 ± 4	5	16	35	10 ± 4	0	10	23	14 ± 4	4	13	31
Cerebrovascular diseases (I60-I69)	12 ± 5	2	12	26	16 ± 5	4	16	33	10 ± 4	1	10	23	14 ± 4	3	13	29
Cardiorespiratory diseases (I00-J99)	34 ± 10	10	35	62	47 ± 10	23	47	85	29 ± 9	9	29	54	40 ± 9	18	40	75

### Meteorology and air pollution

The descriptions of daily meteorological parameters and air pollutants by time period are shown in Table 2. Daily mean temperature, relative humidity, and barometric pressure also followed seasonal patterns, but each with different directions and magnitudes. Due to the fact that air pollution data was only available for a shorter time period, we couldn't confidently detect a seasonal pattern within this data (see Additional file 1, Figure S2).

**Table 2:** Descriptive statistics of meteorological parameters and air pollutants in the urban area of Beijing by time period

Time period	Meteorological parameter/air pollutant	Mean ± SD	Min	1^st ^Qu	Median	3^rd ^Qu	Max
Warm	Air temperature (°C)	22.6 ± 4.8	6.9	19.8	23.3	26.2	32.1
period	Apparent temperature (°C)	23.6 ± 6.8	4.3	19.2	24.5	29.0	37.7
	Relative humidity (%)	60.5 ± 18.1	10	48	63	74	95
	Barometric pressure (hPa)	005.0 ± 59.5	989	1001	1005	1009	1023
	PM_2.5 _(μg/m^3^) ^a^	105.7 ± 66.8	9.9	53.6	98.4	138.2	436.7
	UFP (number/cm^3^) ^a^	23940 ± 9442	9024	16920	21890	29310	73010
							
Cold	Air temperature (°C)	3.4 ± 6.3	-9.1	-1.5	2.3	8.2	18.7
Period	Apparent temperature (°C)	2.9 ± 5.5	-8.0	-1.3	1.9	6.4	18.1
	Relative humidity (%)	47.8 ± 20.8	12 31	46	63	96	
	Barometric pressure (hPa)	1021.0 ± 65.4	999	1016	1021	1025	1037
	PM_2.5 _(μg/m^3^) ^a^	122.0 ± 94.6	13.9	47.5	90.3	184.4	413.4
	UFP (number/cm^3^) ^a^	30940 ± 10637	13460	24120	29150	35550	76280

a. Particulate air pollution data were available only for the period from March 2004 on.

PM_2.5_: particulate matter (PM) with an aerodynamic diameter <2.5 μm.

UFP: ultrafine particles, particles with an aerodynamic diameter <0.1 μm.

### Regression results

**Figure 2:**
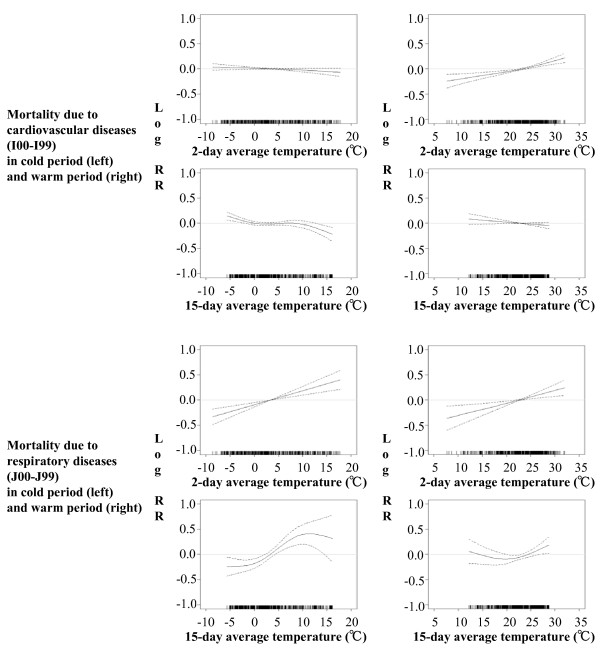
**Exposure-response functions (together with 95% CIs) for two-day and 15-day average temperature and daily mortality of the whole population due to cardiovascular and respiratory diseases in the urban area of Beijing, by warm/cold period**.

Among all the exposure-response functions (Figure 2 and Additional file 1, Figure S3) between two-day or 15-day average temperature and mortality of the whole population due to different causes, only the association between 15-day average temperature and mortality due to respiratory diseases in the warm period showed J-shaped curve (Figure 2), all the other functions could be considered as linear. The exposure-response functions for the elderly population had similar shapes (data not shown).

Based on the AIC, it appeared that 21.3°C was the most appropriate threshold temperature for the one J-shaped relationship. The RRs of mortality associated with mean temperature by time period and age group are shown in Table 3. In the warm period, heat effects were found for two-day average temperature and mortality due to all causes except for ischemic heart diseases. The strongest one was seen for respiratory mortality of the whole population. For elderly people, the RRs were lower for respiratory mortality, higher for cerebrovascular mortality, and almost the same amount for cardiovascular and cardiorespiratory mortality compared to the whole population. Heat effect was also found for 15-day average temperature and respiratory mortality, but with slightly higher effect size for elderly people. In the cold period, heat effects were also found, for both two-day and 15-day average temperature and respiratory mortality, also with higher effect size for the whole population compared to the elderly. Cold effects were found between 15-day average temperature and mortality due to all the other four causes, as well as between two-day average temperature and ischemic heart diseases mortality. The sizes of the effects for elderly people were all lower, although not much, compared to those for the whole population.

**Table 3:** Relative risks (RR, with 95% confidence intervals (CI)) of daily mortality in association with a 5°C increase of 2-day average temperature or a 5°C decrease of 15-day average temperature in the urban area of Beijing, by time period, age group and cause of death

	Warm period		Cold period	
	
	RR (95%CI) per 5°C **increase **of 2-day average temperature	RR (95%CI) per 5°C **decrease **of 15-day average temperature	RR (95%CI) per 5°C **increase **of 2-day average temperature	RR (95%CI) per 5°C **decrease **of 15-day average temperature
Whole population				
Cardiovascular disease (I00-I99)	1.098(1.057,1.140) *	1.040(0.990,1.093)	0.982(0.958,1.007)	1.057(1.022,1.094) *
Respiratory disease (J00-J99)	1.134(1.050,1.224) *	0.937(0.899,0.976) *^a^	1.149(1.078,1.224) *	0.851(0.767,0.944) *
Ischemic heart diseases (I20-I25)	1.020(0.975,1.067)	0.997(0.915,1.087)	0.947(0.914,0.982) *	1.123(1.057,1.193) *
Cerebrovascular diseases (I60-I69) Cardiorespiratory diseases (I00-J99)	1.047(1.000,1.097) *	1.025(0.950,1.106)	0.980(0.954,1.007)	1.036(1.002,1.071) *
	1.114(1.076,1.153) *	1.033(0.968,1.101)	1.009(0.983,1.035)	1.057(1.006,1.111) *
65+ 65+ years				
Cardiovascular disease (I00-I99)	1.093(1.048,1.139) *	1.038(0.978,1.101)	0.994(0.965,1.023)	1.054(1.016,1.093) *
Respiratory disease (J00-J99)	1.080(1.010,1.154) *	0.931(0.890,0.973) *^a^	1.128(1.056,1.204) *	0.887(0.798,0.988) *
Ischemic heart diseases (I20-I25)	1.016(0.968,1.067)	0.978(0.888,1.077)	0.954(0.920,0.990) *	1.116(1.046,1.191) *
Cerebrovascular diseases (I60-I69)	1.064(1.008,1.123) *	1.008(0.928,1.095)	0.999(0.961,1.038)	1.031(0.978,1.087)
Cardiorespiratory diseases (I00-J99)	1.117(1.075,1.160) *	1.010(0.941,1.084)	1.025(0.997,1.054)	1.042(1.001,1.085) *

a. Threshold model for a threshold of 21.3**°**C.

**Figure 3:**
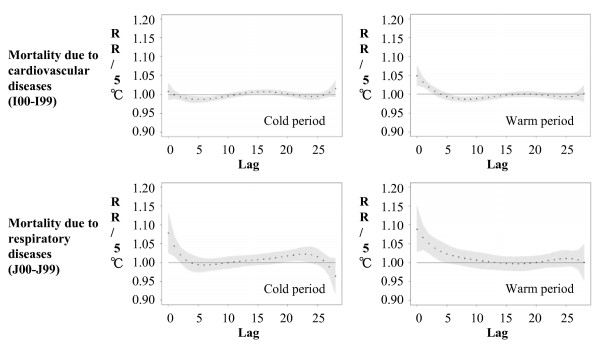
**Relative risks (together with 95% CIs) of mortality of the whole population due to cardiovascular and respiratory diseases in association with a 5°C increase of temperature obtained with polynomial distributed lag models**. Models were estimated with lags up to 29 days using a fifth degree polynomial for the cold period and the warm period. Indicated in each plot are the overall 29-day relative risks.

Figure 3 and Additional file 1, Figure S4 together show polynomial distributed lag curves with daily mean temperature for mortality of the whole population due to all causes. In the warm period, heat effects were always observed within the first five days, whereas a delayed cold effect was observed only for cardiovascular mortality and disappeared with a lag of about two weeks. It is debatable if the described "delayed cold effect" is real or also partly reflects a harvesting effect (mortality displacement) following the heat effect that might have led to an accumulation of premature deaths in the susceptible subpopulation. In the cold period, a heat effect was also observed for respiratory mortality. Apparent one to eight days delayed cold effects were observed for cardiovascular, cerebrovascular and cardiorespiratory mortality, showing no significant following harvesting effect. The polynomial distributed lag curves restricted to the elderly population were similar to the ones for the whole population (data not shown).

### Sensitivity analyses

The exposure-response curves for the whole population obtained by using different values of smoothness for the functions of time trend were quite robust (data not shown). When using different threshold temperatures (21.1, 21.2 and 21.4-21.7°C), the effects of 15-day average temperature on respiratory mortality of the whole population as well as the elderly people in the warm period all became slightly weaker; the size of the effect on elderly people was still higher than the one on the whole population (data not shown). The exposure-response functions for apparent temperature were similar to the ones derived from the mean temperature analyses (data not shown).

The exposure-response curves for the whole population during the shorter period showed almost no change compared to those in Figure 2 as well as in Additional file 1, Figure S3, although the 95%CI of some curves became wider (data not shown). The correlations between temperature and PM_2.5 _as well as UFP by time period are presented in Additional file 1, Table S2. As shown in Additional file 1, Table S3, compared with the effects obtained without adjustment for particle air pollution, there were no relevant changes for the effects of two-day average temperature after controlling for lag 2 of PM_2.5 _or UFP, either in warm or in cold period. The effects of 15-day average temperature on cardiovascular and cardiorespiratory mortality in the warm period dropped and became non-significant after controlling for lag 2 of PM_2.5 _or UFP; while which in the cold period only dropped after controlling for lag 2 of PM_2.5_.

## Discussion

### Summary

We only observed J-shaped association between 15-day average temperature and respiratory mortality in the warm period; the other associations did not diverge from linearity. Immediate heat effects could be seen on every outcome in the warm period, even on respiratory mortality in the cold period; while in the cold period, prolonged cold effects could be seen on every outcome except for respiratory mortality. Previous studies also found immediate heat effects and delayed cold effects [24-26]. The strongest immediate heat effect in the warm period was found in association with respiratory mortality, stronger for the whole population than for elderly people (65 years and older). However, the prolonged heat effect on respiratory mortality, as well as the immediate heat effects on cardiovascular, cerebrovascular and cardiorespiratory mortality in the warm period appeared with similar magnitudes for the two age groups. The strongest cold effect in the cold period was found in association with ischemic heart diseases mortality, with similar effect magnitudes for the whole as well as the elderly population. The prolonged cold effects on cardiovascular, cerebrovascular and cardiorespiratory mortality in the cold period appeared also with similar magnitudes for the two age groups. When considering PM_2.5 _or UFP with lag 2 as confounders, there were no relevant changes for two-day average temperature effects, and a drop in 15-day average temperature effects on cardiovascular and cardiorespiratory mortalities

### Heat effects

For the J-shaped exposure-response function, we found 21.3°C as our most appropriate threshold temperature. Curriero et al. [9] got 19°C to 21°C as "minimum mortality temperature" for New York (NY), Philadelphia (PA), Baltimore (MD), Washington, D.C., U.S., while Ballester et al. [27] got 22°C to 25°C for Valencia, Spain. Those cities are all located on similar latitudes (from 38°54'N to 40°54'N) as Beijing (39°54'N), and "turning-point temperatures" were all close to each other. However, both authors observed V-shaped temperature-mortality functions.

For the convenience of comparison, we re-calculated the percentages based on a 1°C increase in lag 0-1 average temperature in the warm period, which resulted in 2.5% and 1.9% increases in respiratory and cardiovascular mortality, respectively. A study conducted in four Asian cities including Beijing reported by Chung et al. [7] found much higher threshold temperature (31°C) and temperature effect estimates (10.5% and 7.6% per 1°C increase, respectively). The fact that they used daily apparent temperature and also included the entire Beijing (including the suburban area with approximately 4,100,000 inhabitants, the fifth national census in 2000, http://www.stats.gov.cn/tjsj/ndsj/renkoupucha/2000pucha/pucha.htm) as their study area might be the reasons for the differences. However, Almeida et al. [28] reported estimates which are more comparable to ours in their study in Lisbon (38°42'N), Portugal (1.7% and 2.4% per 1°C increase, respectively) using also daily apparent temperature. There are also studies pointing out heat effect on ischemic mortality [26,29], which has not been found in our results.

Similar to the present study, several authors [7,27,30,31] observed greater effect on respiratory mortality than on cardiovascular mortality. In our dataset, daily death counts due to chronic lower respiratory diseases accounted for approximately half of respiratory diseases. However, our explorative analysis revealed that the magnitude of effect on chronic lower respiratory diseases mortality was as high as 98% of respiratory diseases mortality (data not shown). This may reflect that health status of people suffering from chronic respiratory diseases rapidly deteriorates during hot periods [31], which should be kept in mind and considered as priority when setting up preventive strategies during heat events.

After an analysis within the warm period, we found that the order (April to September in 2003 is the 1^st ^warm period, April to September in 2004 is the 2^nd ^one, and April to August in 2005 is the 3^rd ^one.) showed no significant interaction with two-day average temperature, and therefore indicates that there was no heat effect modification by potential population adaption to heat or by possible increasing prevalence of air-conditioning year by year.

### Cold effects

After re-calculation of the cold effects in the cold period, we found 1.1% and 2.3% increases in cardiovascular and ischemic heart disease mortality of our whole study population associated with 1°C decrease in 15-day average temperature, respectively. Analitis et al. [13] reported a higher estimate (a 1°C decrease induced a 1.7% increase in cardiovascular mortality in cold seasons) within the PHEWE project, which might be attributed to their use of a 16-day average of minimum apparent temperature. Moreover, the PHEWE project included very cold northern cities such as Helsinki and Stockholm. However, the Eurowinter study [11] found that people in cold regions such as Finland did not experience more winter excess mortality than people in mild regions such as London; Donaldson et al. [32] observed no excess ischemic heart disease mortality as temperature fell from 10.2°C to -48.2°C in Yakutsk, eastern Siberia. Both findings reflect the possibility of population acclimatization to climate and maybe also to future climate changes. However, the associations of mortality with environmental temperatures are also strongly modified by behavioural and social factors (e.g. clothing, housing conditions) [33]. In Beijing, the residential heating system works every year from November 15^th ^to next March 15^th^, regardless of outside temperatures. However, we found no interaction between heating and 15-day average temperature after an analysis within the cold period, showing that residential heating didn't modify the cold effect. This might also reflect that the study population exposed themselves to outdoor temperature although they probably spent a lot of time indoors.

Our study showed effects of increasing temperature on respiratory mortality even during cold season. This is contrary to our initial hypothesis, although the same situation has been observed by Kunst et al. [34] in The Netherlands. We therefore investigated the exposure-response functions between 2-day or 15-day average temperature and mortality due to influenza and pneumonia (J10-J18) and chronic lower respiratory diseases (J40-J47) (data not shown). Interestingly, we observed different effects regarding the two mortality categories. Whereas a decrease in temperature was associated with an increase in mortality due to influenza and pneumonia (as expected), we found opposite effects for mortality due to chronic lower respiratory diseases. In a previous study, Hampel et al. [35] have reported differences in the associations between a temperature decrease and several blood markers of inflammation and coagulation in patients with coronary diseases and patients with pulmonary diseases. They hypothesized that there might be different disease patterns as well as patient characteristics and medication responsible for the observed differences in the effects. Nevertheless, although we have no hint of a higher misdiagnosis for respiratory deaths than for deaths due to other causes, we cannot rule out this possibility.

### Mechanism

Some studies [36,37] have shown that respiratory mortality increases more for individuals of 65 years and older compared to the general population when air temperature increases. One possible explanation is that aged people, especially COPD patients, are likely to have bad excess heat dissipation through circulatory adjustment. The heat stress increases their risk of developing pulmonary vascular resistance secondary to peripheral pooling of blood or hypovolemia [38]. However, our results didn't show a higher risk for respiratory mortality in individuals of 65 years and older, although half of the respiratory deaths in our study period were due to chronic lower respiratory diseases (J40-J47, mainly COPD, data not shown). It can be speculated that aged people in Beijing pay more attention and expose themselves less to heat. Possible mechanisms through which high temperature increases cardiovascular mortality include enlarged skin vessels and facilitated sweat, leading to falling blood pressure, increased cardiac work load and loss of fluid and salt, further leading to haemoconcentration [39], a "thrombosis promoting" state. The activation of coagulation and inhibition of fibrinolysis lead to diffuse microvascular thrombosis. Besides, heat-induced release of interleukin (IL)-1 or IL-6 into systemic circulation results in damage and hyperactivation of endothelial cells.

When temperature decreases, the cold receptors in skin are stimulated, the sympathetic nervous system regulates the catecholamine level to increases [40] and then the skin vessels constrict to reduce heat loss. Blood pressure increases consequently, and approximately 1l of blood plasma is shifted from skin and legs to central body parts, then removed by urine or shifted to extra-cellular space. The shift of blood plasma leads to haemoconcentration, then the concentrations of red and white blood cells, platelets, certain clotting factors, cholesterol and fibrinogen, as well as blood viscosity all go up, promoting clotting and thrombosis. Moreover, protein C, which is an anticoagulant, moves out to the extra-cellular space with blood plasma. The rise of blood pressure may lead to oxygen deficiency in the cardiac muscle which might induce myocardial ischemia or arrhythmias. If the rise of blood pressure is too sudden, there is the possibility of vascular spasm and a rupture of an atherosclerotic plaque that induces a thrombus [39,41-43].

### Strengths and limitations

This study was based on a population as large as seven million inhabitants, among which the daily cardiorespiratory death count reached 40. This ensured the statistical power of the analysis. Moreover, we did sensitivity analyses by including PM_2.5 _or UFP concentration levels as confounders. Both PM_2.5 _and UFP [44] have been shown to be associated with mortality. As Beijing is known as one of the most polluted cities of the world, controlling for these two air pollutants was an important strength of the present study. Some other studies [36,45,46] also considered PM_2.5_, PM_10 _or black smoke as confounders.

However, there are also limitations of the present study. Firstly, we got both, the meteorological and the air pollution data from only one monitoring station, which may lead to misclassification of the exposure level. This misclassification is non-differential and should bias the effect estimates towards the null. However, further data on daily meteorological parameters from an internet service (Weather Underground 2011) was obtained for a station located in the center of Beijing. Data from the two sources showed a good agreement (Pearson correlation coefficient >0.99 for air temperature). Secondly, ozone is a potentially important confounder to heat effect, but we had no such data for a sensitivity analysis.

## Conclusions

Our results add to the evidence that both increases and decreases in air temperature are associated with an increased risk of cardiovascular mortality. The effects of heat were immediate while the ones of cold became predominant with longer time lags. The increase in air temperature also immediately elevated the risk for respiratory mortality.

## List of abbreviations

PDL models: Polynomial Distributed Lag models; 95%CI: 95% Confidence Interval; ICD-10: International Classification of Diseases 10th Revision; PM_2.5_: Particulate matter with an aerodynamic diameter <2.5 μm; UFP: Particulate matter with an aerodynamic diameter <0.1 μm; DOW: Day of the week; PACF: Partial Autocorrelation Function; DF: Degree of freedom; GCV: Generalized Cross Validation; RR: Relative Risk; COPD: Chronic Obstructive Pulmonary Disease; IL: Interleukin.

## Competing interests

The authors declare that they have no competing interests.

## Authors' contributions

LL performed the statistical analyses and drafted the manuscript. SB guided the statistical analyses and the interpretation of the results substantially, and revised the manuscript substantially and critically. XP was substantially involved in the acquisition of mortality, meteorological and air pollution data, and revised the manuscript critically. UF and AML were involved in the acquisition of respiratory mortality data, and revised the manuscript critically. AW was involved in the acquisition of air pollution data, and revised the manuscript critically. SvK guided the statistical analyses and revised the manuscript critically. HEW was substantially involved in the study design and revised the manuscript critically. AP was substantially involved in the study design, and guided the interpretation of the results, and revised the manuscript critically. AS guided the statistical analyses and the interpretation of the results substantially, and revised the manuscript substantially and critically. All authors read and approved the final manuscript.

## Supplementary Material

Additional file 1 **This file contains three additional tables and four additional figures to the manuscript. **They are: - Additional file, Table S1. Confounders included in each base model - Additional file, Table S2. Correlations between air temperature and PM_2.5 _as well as UFP in the urban area of Beijing - Additional file, Table S3. Relative risks (RR, with 95% confidence intervals (CI)) of daily mortality by cause of death and time period in association with a 5°C increase of 2-day average temperature or 5°C decrease of 15-day average temperature in the urban area of Beijing, before and after adjusting for PM_2.5 _or UFP (linearly with the same moving averages as the temperature term, or linearly with lag 2) in the confounder model - Additional file, Figure S1. Daily death counts by cause of death and age group - Additional file, Figure S2. Daily mean air temperature, relative humidity, barometric pressure, and concentration of PM_2.5 _and UFP - Additional file, Figure S3. Exposure-response relationships (together with 95% confidence intervals) for 2-day and 15-day average temperatures and daily mortality of the whole population due to ischemic heart diseases, cerebrovascular diseases and cardio-respiratory diseases in the urban area of Beijing, by time period - Additional file, Figure S4. Relative risks (together with 95% confidence intervals) of mortality of the whole population due to ischemic heart diseases, cerebrovascular diseases and cardiorespiratory diseases in association with a 5°C increase of temperature obtained with polynomial distributed lag models. Models were estimated with lags up to 29 days using a 5th degree polynomial for the cold period and the warm period. Indicated in each plot are the overall 29-day relative risks Click here for file
