# Supplementary material for: Associations between air temperature and cardio-respiratory mortality in the urban area of Beijing, China: a time-series analysis
Source: Environ Health. 2011 May 25;10:51. doi: 10.1186/1476-069X-10-51 (PMC3129291; doi:10.1186/1476-069X-10-51)
Supplement: Additional file 1 — This file contains three additional tables and four additional figures to the manuscript. They are: - Additional file, Table S1. Confounders included in each base model - Additional file, Table S2. Correlations between air temperature and PM2.5 as well as UFP in the urban area of Beijing - Additional file, Table S3. Relative risks (RR, with 95% confidence intervals (CI)) of daily mortality by cause of death and time period in association with a 5°C increase of 2-day average temperature or 5°C decrease of 15-day average temperature in the urban area of Beijing, before and after adjusting for PM2.5 or UFP (linearly with the same moving averages as the temperature term, or linearly with lag 2) in the confounder model - Additional file, Figure S1. Daily death counts by cause of death and age group - Additional file, Figure S2. Daily mean air temperature, relative humidity, barometric pressure, and concentration of PM2.5 and UFP - Additional file, Figure S3. Exposure-response relationships (together with 95% confidence intervals) for 2-day and 15-day average temperatures and daily mortality of the whole population due to ischemic heart diseases, cerebrovascular diseases and cardio-respiratory diseases in the urban area of Beijing, by time period - Additional file, Figure S4. Relative risks (together with 95% confidence intervals) of mortality of the whole population due to ischemic heart diseases, cerebrovascular diseases and cardiorespiratory diseases in association with a 5°C increase of temperature obtained with polynomial distributed lag models. Models were estimated with lags up to 29 days using a 5th degree polynomial for the cold period and the warm period. Indicated in each plot are the overall 29-day relative risks [file 1476-069X-10-51-S1.DOC]

**Additional file, Table S1. Confounders included in each base model ***

**Population Confounders**

**and Outcome**

**trend season day of the week public relative barometric other**

**(DOW) holiday humidity pressure**

**Whole population**

**Cardiovascular** penalized dummy dummy penalized

**diseases (I00-I99)** spline variable variable spline

**Respiratory** penalized dummy dummy penalized

**disease (J00-J99)** spline variable variable spline

**Ischemic heart** penalized dummy dummy penalized

**diseases (I20-I25)** spline variable variable spline

**Cerebrovascular** penalized dummy dummy penalized death count due to cereborvascular diseases

**diseases (I60-I69)**  spline variable variable spline of lag 1, linear

**Cardiorespiratory** penalized dummy dummy penalized

**disease (I00-J99)**  spline variable variable spline

**65+ years**

**Cardiovascular** penalized dummy dummy penalized

**diseases (I00-I99)** spline variable variable spline

**Respiratory** penalized dummy dummy penalized

**disease (J00-J99)** spline variable variable spline

**Ischemic heart** penalized dummy dummy penalized

**diseases (I20-I25)** spline variable variable spline

**Cerebrovascular** penalized dummy dummy penalized death count due to cereborvascular diseases

**diseases (I60-I69)**  spline variable variable spline of lag 1, linear

**Cardiorespiratory** penalized dummy dummy dummy penalized

**disease (I00-J99)**  spline variable variable variable spline

***** We used the same confounder models for the warm and cold periods; for the sensitivity analysis we only re-adjusted the DF for trend in every model,

because of less days, on which the air pollution data was available.

**Additional file, Table S2. Correlations between air temperature and PM_2.5_ as well as UFP in the urban area of Beijing**

**PM_2.5_ (μg/m^3^)** **UFP (number/cm^3^)**

**Warm period**

**Air temperature (℃)**  0.222 -0.297

**PM_2.5_ (μg/m^3^)**  -0.348

**Cold period**

**Air temperature (℃)**  0.092 -0.028

**PM_2.5_ (μg/m^3^)**  -0.417

**Additional file, Table S3. Relative risks (RR, with 95% confidence intervals (CI)) of daily mortality by cause of death and time period in association with a 5°C increase of 2-day average temperature or 5°C decrease of 15-day average temperature in the urban area of Beijing , before and after adjusting for PM_2.5_ or UFP (linearly with the same moving averages as the temperature term, or linearly with lag 2) in the confounder model**

Warm period Cold period

RR (95%CI) per 5°C RR (95%CI) per 5°C RR (95%CI) per 5°C RR (95%CI) per 5°C

**increase** of 2-day **decrease** of 15-day **increase** of 2-day **decrease** of 15-day

average temperature average temperature average temperature average temperature

The whole population

**No adjustment for air pollutants**

Cardiovascular disease (I00-I99) 1.066(1.016,1.118) *  1.192(1.051,1.352) * 0.969(0.945,0.994) * 1.101(1.003,1.209) *

Respiratory disease (J00-J99) 1.079(0.992,1.174) 0.940(0.891,0.991) *^a^ 1.100(0.995,1.216) 0.930(0.769,1.125)

Ischemic heart diseases (I20-I25) 0.999(0.941,1.061)  1.064(0.964,1.175) 0.981(0.945,1.018) 1.004(0.947,1.066)

Cerebrovascular diseases (I60-I69) 1.069(1.007,1.136) * 1.033(0.936,1.141) 0.978(0.941,1.016) 1.018(0.974,1.065)

Cardiorespiratory diseases (I00-J99) 1.083(1.036,1.133) * 1.103(1.002,1.215) * 1.009(0.983,1.035) 1.057(1.006,1.111) *

**PM_2.5_ (linearly with lag 2)**

Cardiovascular disease (I00-I99) 1.082(1.024,1.144) * 1.068(0.991,1.150) 0.982(0.930,1.037) 1.041(0.944,1.147)

Respiratory disease (J00-J99) 1.079(0.986,1.181) 0.929(0.877,0.984) *^a^ 1.105(0.963,1.267) 0.907(0.702,1.172)

Ischemic heart diseases (I20-I25) 1.003(0.939,1.070) 1.029(0.938,1.129) 0.962(0.920,1.005) 1.021(0.946,1.103)

Cerebrovascular diseases (I60-I69) 1.072(1.004,1.144) * 1.000(0.899,1.113) 1.001(0.956,1.048) 1.002(0.953,1.054)

Cardiorespiratory diseases (I00-J99) 1.105(1.050,1.164) * 1.069(0.987,1.158) 1.010(0.950,1.074) 1.013(0.898,1.143)

**UFP (linearly with lag 2)**

Cardiovascular disease (I00-I99) 1.080(1.027,1.136) * 1.052(0.990,1.118) 0.970(0.944,0.998) * 1.118(1.006,1.242) *

Respiratory disease (J00-J99) 1.078(0.988,1.177) 0.933(0.880,0.989) *^a^ 1.115(1.000,1.244) * 1.049(0.958,1.148)

Ischemic heart diseases (I20-I25) 1.020(0.957,1.086) 1.017(0.929,1.113) 0.971(0.933,1.011) 1.023(0.961,1.190)

Cerebrovascular diseases (I60-I69) 1.073(1.008,1.142) * 1.016(0.914,1.129) 0.987(0.946,1.029) 1.005(0.957,1.055)

Cardiorespiratory diseases (I00-J99) 1.094(1.045,1.146) * 1.064(0.990,1.142) 1.010(0.963,1.059) 1.077(0.970,1.194)

a. Threshold model for a threshold of 21.3**°**C.

**Additional file, Figure S1. Daily death counts by cause of death and age group**

**Additional file, Figure S2.** **Daily mean air temperature, relative humidity, barometric pressure, and concentration of PM_2.5_**† **and UFP**†


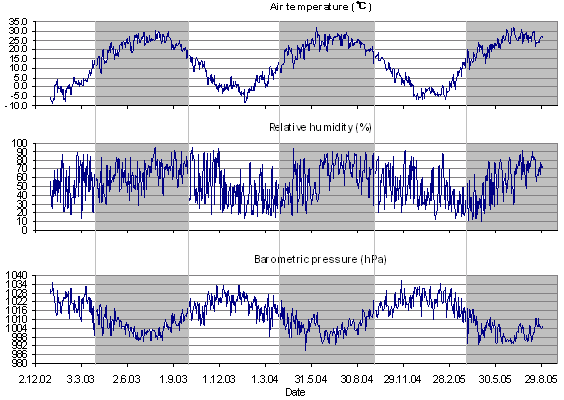


*
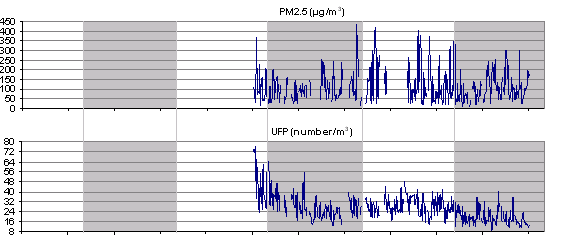
*

*
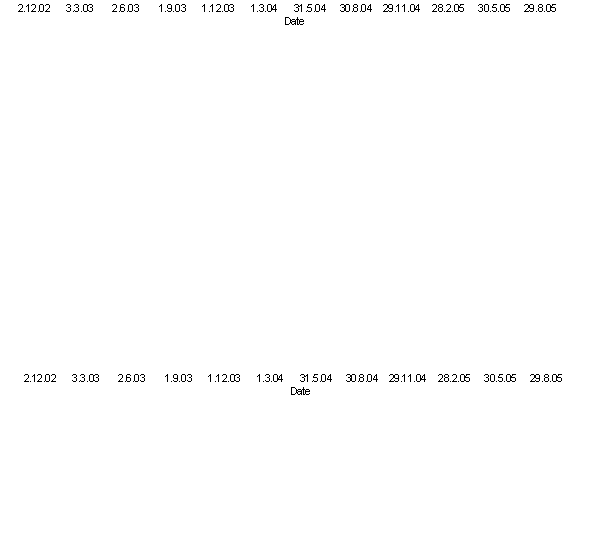
*

*Cold period Cold period Cold period*

*Warm period Warm period Warm period*

† Particle data were available only for the period March 2004 until August 2005.

**Additional file, Figure S3. Exposure-response relationships (together with 95% confidence intervals) for 2-day and 15-day average temperatures and daily mortality of the whole population due to ischemic heart diseases, cerebrovascular diseases and cardio-respiratory diseases in the urban area of Beijing, by time period**

**Additional file, Figure S4. Relative risks (together with 95% confidence intervals) of mortality of the whole population due to ischemic heart diseases, cerebrovascular diseases and cardiorespiratory diseases in association with a 5°C increase of temperature obtained with polynomial distributed lag models. Models were estimated with lags up to 29 days using a 5th degree polynomial for the cold period and the warm period. Indicated in each plot are the overall 29-day relative risks**
